# Beyond HIV: investigating key drivers of metabolic syndrome in midlife women with HIV in the INSTI era

**DOI:** 10.1186/s12981-026-00913-1

**Published:** 2026-07-01

**Authors:** M. Poliseno, G. Brindicci, D. Capruzzi, A. Cianciaruso, N. Colavito, V. Giliberti, V. Lobalsamo, G. Metrangolo, E. Milano, L. Perrucci, C. R. Santoro, A. Vigna, F. Di Gennaro, A. Saracino

**Affiliations:** 1 Clinic of Infectious Diseases, Department of Precision and Regenerative Medicine and Jonian Area (DiMePre-J), University of Bari “Aldo Moro”, Piazza Giulio Cesare 11, 70124 Bari, Italy; 2 University of Bari “Aldo Moro”, Piazza Umberto I, 70121 Bari, Italy

**Keywords:** HIV, ART, Metabolic syndrome, INSTI, TAF, Women with HIV

## Abstract

**Background:**

The contribution of lifestyle factors compared with HIV infection and antiretroviral therapy (ART)-related determinants to Metabolic Syndrome (MetS) in Women with HIV (WWH) remains uncertain.

**Objectives:**

To evaluate the prevalence and determinants of MetS in the era of Integrase Strand Transfer Inhibitors (INSTI).

**Design:**

Monocentric retrospective cohort study.

**Methods:**

We analyzed 297 WWH to assess the prevalence of MetS and its association with HIV-related and lifestyle factors.

**Results:**

MetS was diagnosed in 80 women (26%). Those with MetS showed lower CD4 nadir, longer HIV and ART duration, and greater exposure to protease inhibitors and INSTI, but none were independently associated with MetS or its components. Notably, lifestyle-related factors showed relatively strong associations with the study outcomes: physical activity was protective (OR 0.10, 95% CI 0.02–0.44), whereas physically demanding work increased risk (OR 6.58, 95% CI 2.03–26.26).

**Conclusions:**

Lifestyle-related factors appeared to show stronger associations with MetS risk compared to HIV-related variables, underscoring the importance of systematic lifestyle assessment and targeted interventions in women with HIV.

**Supplementary Information:**

The online version contains supplementary material available at https://doi.org/10.1186/s12981-026-00913-1.

## Introduction

Metabolic syndrome (MetS) is a cluster of interrelated risk factors that, according to the United States National Cholesterol Education Program/Adult Treatment Panel III (USNCEP ATP-III) criteria, [1] includes at least three among the following features: abdominal obesity, low levels of high-density HDL-cholesterol, insulin resistance, and hypertension. It is well documented that MetS is a strong predictor of type 2 diabetes, cardiovascular disease, renal disease, and premature mortality [2], significantly contributing to increasing the economic burden with increased medical and health care costs [3].

Among People With HIV (PWH), recent data report almost twice the risk of developing MetS than the general population [4]. The mechanisms underlying MetS in people living with HIV are likely multifactorial and involve an interplay between traditional metabolic risk factors and HIV-related factors, including chronic immune activation, persistent inflammation, and potential long-term metabolic effects of antiretroviral therapy (ART) [5]. These HIV-related mechanisms act in conjunction with established risk factors for non-communicable diseases (NCDs), such as advancing age, tobacco use, excessive alcohol intake, unhealthy dietary habits, and physical inactivity [6], together contributing to the overall metabolic risk profile observed in this population.

Moreover, among Women with HIV (WWH), a combination of biological, hormonal, cultural, and social factors further increases the risk of MetS compared to both women without HIV and men living with HIV [7–9].

Nevertheless, the relative contribution of lifestyle-related factors versus HIV- and ART-related determinants to the development of MetS has been insufficiently explored in the modern era of Integrase Strand Transfer Inhibitors (INSTI). In this setting, ART initiation has dramatically changed in both timing—being now recommended soon after diagnosis, thereby reducing virus-driven inflammation [10]- and regimen composition, with current therapies showing a more favorable impact on lipid profiles compared to older antiretroviral drugs, particularly those from the Non-Nucleoside Reverse Transcriptase Inhibitors (NNRTIs) class [11]. In addition, evidence on the burden of individual MetS components is limited [9], and it remains unclear which features are most prevalent and contribute most to MetS development according to HIV status and ART exposure.

Our study aims to assess the prevalence and risk factors for the development of MetS and its individual components in a cohort of middle-aged WWH and to evaluate the relative contributions of lifestyle-related versus HIV- and modern ART-related determinants on its development.

## Methods

This study aimed to describe the prevalence of MetS, defined according to the NCEP-ATP III 2005 criteria [1], and its diagnostic components in a cohort of WWH receiving stable ART. We also sought to evaluate the relative contributions of lifestyle-related factors and HIV/ART-related variables to the likelihood of developing MetS and its individual components.

All women in active follow-up (defined as at least one clinical visit and/or blood test in the previous year) at the HIV outpatient clinic of the University Hospital of Bari were eligible for inclusion, provided they gave written informed consent. The center is located in the main hospital of the Apulia region, within the Metropolitan City of Bari, a predominantly urban area with approximately 1.2 million inhabitants. The HIV outpatient clinic provides care for a cohort of approximately 1400 PWH , within a large tertiary referral setting. Between January 1 and July 30, 2025, all participants underwent a structured clinical evaluation for MetS and a standardized interview to collect information on menopausal status, occupational and physical activity, lifestyle habits, and family history of cardiometabolic comorbidities. Biochemical parameters (HDL- and LDL-cholesterol, triglycerides, and fasting glucose) were extracted from the Institutional electronic database.

A descriptive analysis was performed, using Chi-square and Mann–Whitney U-tests to compare WWH with and without MetS. Bivariate and multivariate logistic regression models were used to assess factors associated with MetS. Multivariable models were adjusted for chronological and reproductive age, physical activity, work type, smoking and alcohol habits, HIV-related factors (mode of transmission, nadir CD4, infection, and ART duration), and ART exposure to Tenofovir Alafenamide (TAF), Protease Inhibitors (PIs), and INSTI. Odds Ratios (O.R.) with 95% Confidence Intervals (C.I.) were estimated.

Models were applied both with MetS as the outcome and with single components (hypertension, hyperglycemia, HDL-cholesterol (HDL-Chol) < 50 mg/dL, triglycerides (TGL) > 150 mg/dL, waist circumference > 88 cm). Statistical analyses were performed using Jamovi v2.6.44 [12]. The study was approved by the Local Ethics Committee on June 16, 2025 (Prot. 2241/CEI).

## Results

A total of 297 WWH, ranging from 21 to 90 years of age, agreed to have their data included for research purposes out of 320 women currently followed at our outpatient clinic. Notably, the cohort included predominantly middle-aged to older WWH with a long history of HIV infection and ART (Table 1).

**Table 1:** Characteristics of 297 Women With HIV (WWH) stratified according to Metabolic Syndrome (MetS) diagnosis

Variables	WWH without MetS (N = 217)	WWH with MetS (N = 80)	Total (N = 297)	*p*-value§
Non Italian Nationality, n (%)	26 (12)	11 (14)	37 (12)	0.68
Age, years, median (IQR)	56 (49–60)	60 (54–65)	56 (50–61)	** < 0.001**
< 40 years, n (%)	28 (13)	5 (6)	33 (11)	** < 0.001**
40–49 years, n%	27 /12)	3 (4)	30 (10)	
50–59 years, n (%)	107 (49)	30 (37)	137 (46)	
≥ 60 years, n (%)	55 (25)	42 (52)	97 (33)	
HIV transmission route, n (%)				
Heterosexual contact	128 (59)	40 (50)	168 (56)	0.26
PWID	9 (4)	7 (9)	16 (5)	
Blood products	3 (1)	1 (1)	4 (1)	
Vertical	4 (2)	0 (0)	4 (1)	
Unknown	73 (34)	32 (40)	105 (35)	
HBsAg positive, n (%)	8 (4)	3 (4)	11 (4)	0.99
HCV-Ab positive, n (%)	29 (13)	14 (18)	43 (15)	0.39
CD4 nadir, cells/mL, median (IQR)	193 (107–289)	119 (70–123)	183 (90–274)	**0.003**
CD4 nadir < 200 cells/mL, median (IQR)	109 (53)	50 (68)	159 (57)	**0.035**
HIV RNA zenith, cp/mL, median (IQR)	81,500 (21,750–285,000)	88,000 (17,000–287,000)	86,000 (21,000–310,000)	0.75
AIDS-defining conditions at baseline, n (%)	32 (15)	18 (23)	50 (17)	0.11
History of HIV infection, years, median (IQR)	22 (14–31)	27 (21–33)	25 (15–32)	**0.006**
≥ 10 years, n (%)	188 (87)	74 (92)	262 (88)	0.16
History of ART, years, median (IQR)	20 (12–27)	25 (20–28)	22 (13–28)	** < 0.001**
≥ 10 years, n (%)	173 (809)	71 (89)	244 (82)	0.07
Undetectable, n (%)	172 (79)	65 (81)	237 (80)	0.70
Current ART regimen, n (%)				0.60
2 NRTI + NNRTI	38 (17)	18 (22)	56 (19)	
2 NRTI + PI	6 (3)	6 (7)	12 (4)	
2 NRTI + INSTI	71 (34)	27 (34)	98 (33)	
NRTI + INSTI	50 (23)	13 (16)	63 (21)	
NNRTI + INSTI	42 (19)	11 (14)	53 (18)	
Other	10 (5)	5 (6)	15 (5)	
Currently on STR, n (%)	176 (81)	65 (81)	241 (81)	0.98
Currently on LA ART regimen, n (%)	10 (5)	0 (0)	10 (3)	0.05
Currently on 2DR, n (%)	100 (46)	29 (36)	129 (43)	0.13
Currently on TAF-based regimens, n (%)	87 (40)	38 (47)	60 (20)	0.25
Currently on TDF-based regimens, n (%)	26 (12)	12 (15)	38 (12)	0.49
History of TAF, n (%)	149 (70)	77 (52)	206 (70)	0.60
Duration of TAF use, years, median (IQR)	3 (0–6)	4 (0–7)	4 (0–7)	0.23
≥ 5 years, n (%)	74 (34)	33 (41)	107 (36)	0.25
History of PI, n (%)	142 (67)	60 (76)	202 (70)	**0.009**
Duration of PI use, years, median (IQR)	3 (0–9)	7 (0–14)	4 (0–12)	**0.008**
≥ 5 years, n (%)	130 (62)	32 (43)	162 (57)	**0.005**
≥ 10 years, n (%)	54 (25)	33 (41)	97 (29)	**0.006**
History of INSTI, n (%)	169 (78)	57 (61)	226 (76)	0.23
Duration of INSTI use, years, median (IQR)	6 (2–9)	7 (3–9)	6 (2–9)	0.44
≥ 5 years, n (%)	112 (52)	42 (54)	154 (53)	0.79
≥ 10 years, n (%)	27 (13)	16 (21)	43 (15)	**0.009**

^§^Chi-square Test/Mann–Whitney U-test as appropriate

IQR: Inter Quartile Range, WWH: Women With HIV, MetS: Metabolic Syndrome, HIV: Human Immunodeficency Virus, PWID: People Who Injected Drugs, HBsAg: Hepatitis B virus Surface Antigen, HCVAb: Hepatitis C Virus Antibodies, AIDS: Acquired Immunodeficiency Syndrome, ART: Antiretroviral Therapy, NRTI: Nucleoside Reverse Transcriptase Inhibitor, LA ART: Long Acting Antiretroviral Therapy, STR: Single Tablet Regimen, TAF: Tenofovir Alafenamide, TDF: Tenofovir Disoproxil, 2DR: Two Drug Regimen, PI: Protease Inhibitors, INSTI: Integrase Strand Transfer Inhibitors

Overall, 141 WWH (55%) had a waist circumference > 88 cm, 130 (46%) had blood pressure ≥ 130/80 mmHg, 101 (34%) had levels of HDL-cholesterol < 50 mg/dL, and 58 (20%) had fasting glucose levels > 100 mg/dL.

MetS was diagnosed in 80 women, with a prevalence of 26%. Of these, 62 (80%) were postmenopausal and 14 (18%) premenopausal. Women with MetS had a slightly older age at menopause (52 vs 51 years, p = 0.036) and longer menopausal duration (8 [InterQuartile Range, IQR 4–15] vs 4 [IQR 0–10] years) compared to those without MetS. In the MetS group, median body weight was 68 kg (IQR 73–82), waist circumference 94 cm (IQR 90–106), TGL 130 mg/dL (IQR 101–165), fasting glucose 101 mg/dL (IQR 88–113), HDL- Chol 46 mg/dL (IQR 40–50), and LDL-Cholesterol (LDL Chol) 107 mg/dL (IQR 80–137). WWH with MetS were older, had lower CD4 counts at diagnosis, and longer histories of HIV and ART. Although current ART regimens did not differ between groups, cumulative exposure to PIs and INSTI was significantly longer among participants with MetS.

Family history of NCDs was frequent: 45 WWH (62%) reported dyslipidemia, 22 (31%) ischemic heart disease, and 20 (28%) diabetes mellitus, all more prevalent than in WWH without MetS. Alcohol abuse was rare (12% vs 11%, p = 0.90), while smoking was common (47% vs 48%, p = 0.87) in both groups. Most WWH with MetS (73%) held arduous jobs (farm/factory laborer, domestic worker, healthcare assistant, cleaner, nurse), and only 7 (9%) engaged in physical activity.

Risk factor analysis showed a higher O.R. of MetS with arduous work (OR 6.58, 95% C.I. 2.03–26.26, p = 0.003) and a lower O.R. with physical activity (O.R. 0.10, 95% C.I. 0.02–0.44, p = 0.005). (Table 2). In separate MetS components, physical activity reduced the likelihood of waist circumference > 88 cm (O.R. 0.30, 95% C.I. 0.11–0.77, p = 0.015) and hyperglycemia (O.R. 0.19, 95% C.I. 0.04–0.72, p = 0.026). Conversely, hyperglycemia was more likely in women on INSTI for > 10 years (O.R. 5.73, 95% C.I. 1.33–27.62, p = 0.02). Menopause significantly increased the risk of hypertension (O.R. 5.44, 95% C.I. 1.50–24.18, p = 0.015). People Who Injected Drugs (PWID) showed markedly higher odds of hypertriglyceridemia (O.R. 14.39, 95% C.I. 1.49–176.13, p = 0.02). Both family history of dyslipidemia (O.R. 4.06, 95% C.I. 1.56–11.61, p = 0.006) and arduous work (O.R. 4.78, 95% C.I. 1.83–13.73, p = 0.002) independently increased the risk of having low HDL-cholesterol levels. The full associations between variables and the individual components of metabolic syndrome are provided in Tables S1 to S5 in Supplementary Materials.

**Table 2:** Bivariate and multivariate logistic regression analysis of factors associated with MetS.

Covariates	Bivariate O.R. (95% C.I., *p*-value)	Multivariate O.R. (95% C.I., *p*-value)
Age ≥ 60 years	2.13 (0.89–5.12, p = 0.09)	–
Menopause*	1.57 (0.60–4.65, p = 0.38)	–
Physical activity **	0.19 (0.04–0.59, p = 0.01)	**0.10 (0.02–0.44, p = 0.005)**
Arduous work***	4.11 (1.65–11.33, p = 0.004)	**6.58 (2.03–26.26, p = 0.003)**
Active/former smoker	0.95 (0.40–2.22, p = 0.90)	–
Alcohol abuse****	1.08 (0.15–5.01, p = 0.93)	–
Dyslipidemia (family history)	2.02 (0.86–4.92, p = 0.11)	–
PWID	5.28 (0.83–41.78, p = 0.07)	–
≥ 10 Years of HIV	2.00 (0.50–13.39, p = 0.38)	–
≥ 10 Years of ART	2.81 (0.73–18.54, p = 0.19)	–
CD4 nadir < 200 cells/mm3	1.77 (0.74–4.49, p = 0.21)	–
≥ 5 Years of TAF use	1.17 (0.48–2.79, p = 0.72)	–
≥ 10 Years of PI use	1.83 (0.72–4.52, p = 0.20)	–
≥ 10 Years of INSTI use	1.20 (0.31–3.87, p = 0.77)	–

^*^ Defined as the absence of menstruation for more than 12 months, as reported by patients

^**^ Engagement in any leisure-time physical exercise or activity performed at least once per week for a minimum duration of 60 consecutive minutes as reported by patients

^***^More than 1 standard drink per day (i.e., > 10–12 g of pure ethanol per day) as reported by patients

^****^ Farm laborer, domestic worker, factory laborer, healthcare assistant, nurse, factory worker, hotel/hospital cleaner

O.R.: Odds Ratio, C.I.: Confidence Interval, HIV: Human Immunodeficiency Virus, PWID: People Who Injected Drugs, AIDS: Acquired Immunodeficiency Syndrome, HBsAg: Hepatitis B Surface Antigen, HCVAb: Hepatitis C Virus Antibodies, ART: Antiretroviral Therapy, TAF: Tenofovir Alafenamide, PI: Protease Inhibitors, INSTI: Integrase Strand Transfer Inhibitors

## Discussion

In our cohort of 297 WWH, middle-aged and with a long history of HIV infection and antiretroviral therapy, the prevalence of metabolic syndrome was 26%. Our findings are consistent with published estimates, which, although highly variable depending on historical period, geographic region, age, and diagnostic criteria, report a recent pooled prevalence of approximatively 25% among people with HIV [5].

Specifically, evidence in WWH remains limited and mostly derives from the pre-INSTI era. For instance, in 2008, the Women's Interagency HIV Study reported a MetS prevalence of 33% in women living with HIV versus 22% in the general female population. To note, hypertriglyceridemia, low HDL cholesterol, and hypertension were the most common MetS components among PWH, whereas increased waist circumference predominated in participants without HIV, suggesting distinct pathophysiological mechanisms [10].

In contrast, our analysis highlighted hypertension, increased waist circumference, and reduced HDL cholesterol as the main MetS components. Recent studies similarly identify increased waist circumference and low LDL cholesterol as predominant features among WWH [7, 10], indicating an evolving role of HIV infection, lifestyle, and ART-related toxicity in MetS development.

Meta-analytic evidence suggests ART exposure increases MetS risk by approximately 1.5-fold [5]. Early ART regimens—including NRTIs (didanosine, stavudine, abacavir), NNRTIs (efavirenz), and PIs (ritonavir, lopinavir, darunavir, atazanavir, nelfinavir, saquinavir)—were associated with dyslipidemia, insulin resistance, and fat redistribution [13–16]. More recent studies, including the START trial [11], indicate that modern ART may have neutral or even beneficial metabolic effects.

In our cohort, more than 70% of women were receiving INSTI-based regimens. While some studies have reported an association between INSTI use and weight gain with potential metabolic consequences [17, 18], this relationship remains controversial, as other investigations have not confirmed a consistent effect and even highlight a potential protective role on INSTI on MetS [19–21]. Moreover, the magnitude of these findings appears to vary according to the specific INSTI molecule and population characteristics. While some reports suggest dolutegravir may slightly increase risk, cabotegravir/rilpivirine injections might be protective, though long-term confirmation is needed [22]. A novelty of our study is that we analyzed not only the impact of the current ART regimen on MetS prevalence—potentially biased by channeling, since patients at perceived high risk for MetS may have been preferentially started on or switched to supposedly protective regimens—but also the cumulative years of exposure to PIs and INSTI, finding that neither class significantly increased MetS risk. However, a definitive interpretation remains challenging and our findings should be interpreted with caution in the context of this ongoing debate.

According to our observations, key drivers of MetS should be sought in daily lifestyle behaviours. Indeed, we observed a protective role of physical activity, consistent with findings in the general population [23]. Conversely, women in physically demanding occupations showed a higher risk of MetS [24].

Finally, unlike previous studies linking reproductive status to MetS risk [13], we highlight no association with chronological age or with menopausal status and MetS.

All these results taken together suggest that, beyond considerations related solely to ART regimen switching, there is a concrete need for individualized counselling and prevention strategies in all WWH.

This study has several limitations. Firstly, given that the incidence of MetS is strongly influenced by socioeconomic and cultural factors, level of healthcare access, and local lifestyle habits, our results should be interpreted within the context of the Italian country and the Apulian region. In addition, the observed associations between physically demanding occupations and leisure-time physical activity may reflect underlying socioeconomic differences, as no direct measures of socioeconomic status (SES) were assessed in this study; therefore, lower SES rather than lifestyle factors alone may partly explain the reported findings.

Moreover, some lifestyle factors potentially related to MetS (e.g., diet, stress, and poor sleep) were not assessed. Finally, it should be underlined that relatively small sample size of our cohort, due to the single-center design of our research, may have affected the statistical significance of some results. Finally, that the lack of a control group of women without HIV limits the interpretability and generalizability of the findings, as it is not possible to determine whether the observed associations between lifestyle factors and metabolic syndrome are specific to WWH or reflect patterns that are also present in the general female population.

Larger multicenter studies on WWH are essential to confirm and strengthen the validity of our findings.

## Supplementary Information

Supplementary Material 1.

Supplementary Material 2.

Supplementary Material 3.

Supplementary Material 4.

Supplementary Material 5.

## Data Availability

The datasets generated and/or analyzed during the current study are not publicly available due to privacy and confidentiality restrictions, but are available from the corresponding author on reasonable request.
