# Supplementary material for: Beyond HIV: investigating key drivers of metabolic syndrome in midlife women with HIV in the INSTI era
Source: AIDS Res Ther. 2026 Jul 1;23:92. doi: 10.1186/s12981-026-00913-1 (PMC13625421; doi:10.1186/s12981-026-00913-1)
Supplement: Supplementary file 1 [file 12981_2026_913_MOESM1_ESM.docx]

**Table S1.** Bivariate and Multivariate Cox Regression Analysis of factors associated with high blood pressure.

| Covariates | Bivariate O.R. (95% C.I., p-value) | Multivariatee O.R. (95% C.I., p-value)§ |
| --- | --- | --- |
| Age≥ 60years | 3.20 (1.46-7.22, p=0.004) | 2.14 (0.76-6.25, p=0.15) |
| Menopause* | 7.89 (2.78-28.55, p<0.001) | **5.44 (1.50-24.18, p=0.01)** |
| Physical activity ** | 0.54 (0.24-1.20, p=0.14) | - |
| Arduous work*** | 1.82 (0.87-3.86, p=0.11) | - |
| Active/former smoker | 1.79 (0.86-3.80, p=0.12) | - |
| Alcohol abuse**** | 3.24 (0.67-23.36, p=0.17) | - |
| Dyslipidemia (family history) | 1.35 (0.65-2.84, p=0.42) | - |
| PWID | 5.17 (0.74-102.84, p=0.15) | - |
| ≥10 Years of HIV | 10.79 (1.99-200.79, p=0.02) | 12.44 (0.63-449.21, p=0.113) |
| ≥10 Years of ART | 4.67 (1.42-21.17, p=0.02) | 0.68 (0.08-6.84, p=0.72) |
| CD4 nadir <200 cells/mm3 | 1.87 (0.88-4.06, p=0.10) | - |
| ≥5 Years of TAF use | 1.16 (0.54-2.50, p=0.69) | - |
| ≥10 Years of PI use | 3.31 (1.41-8.23, p=0.007) | 1.86 (0.63-5.69, p=0.26) |
| ≥10 Years of INSTI use | 1.67 (0.58-5.03, p=0.34) | - |

** Defined as the absence of menstruation for more than 12 months, as reported by patients.*

*** Engagement in any leisure-time physical exercise or activity performed at least once per week for a minimum duration of 60 consecutive minutes as reported by patients.*

****More than 1 standard drink per day (i.e., >10–12 grams of pure ethanol per day) as reported by patients*

****** *Farm laborer, domestic worker, factory laborer, healthcare assistant, nurse, factory worker, hotel/hospital cleaner.*

O.R.: Odds Ratio; C.I.: Confidence Interval; HIV: Human Immunodeficiency Virus; PWID: People Who Inject Drugs; AIDS: Acquired Immunodeficiency Syndrome; HBsAg: Hepatitis B Surface Antigen; HCVAb: Hepatitis C Virus Antibodies; ART: Antiretroviral Treatment; TAF: Tenofovir Alafenamide; PI: Protease Inhibitors; INSTI: Integrase Strand Transfer Inhibitors
