# Supplementary material for: Beyond HIV: investigating key drivers of metabolic syndrome in midlife women with HIV in the INSTI era
Source: AIDS Res Ther. 2026 Jul 1;23:92. doi: 10.1186/s12981-026-00913-1 (PMC13625421; doi:10.1186/s12981-026-00913-1)
Supplement: Supplementary file 2 [file 12981_2026_913_MOESM2_ESM.docx]

**Table S2.** Bivariate and Multivariate Cox Regression Analysis of factors associated with Hypertriglyceridemia

| Covariates | Bivariate O.R. (95% C.I., p-value) | Multivariate O.R. (95% C.I., p-value)§ |
| --- | --- | --- |
| Age≥ 60years | 0.83 (0.27-2.28, p=0.73) | - |
| Menopause* | 1.17 (0.41-3.86, p=0.78) | - |
| Physical activity ** | 0.49 (0.13-1.45, p=0.23) | - |
| Arduous work*** | 2.18 (0.82-6.23, p=0.13) | - |
| Active/former smoker | 1.13 (0.43-2.98, p=0.81) | - |
| Alcohol abuse**** | 1.74 (0.24-8.28, p=0.52) | - |
| Dyslipidemia (family history) | 1.83 (0.70-5.05, p=0.22) | - |
| PWID | 8.65 (1.34-69.45, p=0.02) | **14.39 (1.49-176.13, p=0.02)** |
| ≥10 Years of HIV | 0.70 (0.19-3.33, p=0.61) | - |
| ≥10 Years of ART | 1.00 (0.29-4.65, p=1.00) | - |
| CD4 nadir <200 cells/mm3 | 2.56 (0.91-8.34, p=0.09) | - |
| ≥5 Years of TAF use | 0.88 (0.31-2.35, p=0.80) | - |
| ≥10 Years of PI use | 1.29 (0.42-3.59, p=0.64) | - |
| ≥10 Years of INSTI use | 0.74 (0.11-3.01, p=0.71) | - |

** Defined as the absence of menstruation for more than 12 months, as reported by patients.*

*** Engagement in any leisure-time physical exercise or activity performed at least once per week for a minimum duration of 60 consecutive minutes as reported by patients.*

****More than 1 standard drink per day (i.e., >10–12 grams of pure ethanol per day) as reported by patients*

****** *Farm laborer, domestic worker, factory laborer, healthcare assistant, nurse, factory worker, hotel/hospital cleaner.*

O.R.: Odds Ratio; C.I.: Confidence Interval; HIV: Human Immunodeficiency Virus; PWID: People Who Inject Drugs; AIDS: Acquired Immunodeficiency Syndrome; HBsAg: Hepatitis B Surface Antigen; HCVAb: Hepatitis C Virus Antibodies; ART: Antiretroviral Treatment; TAF: Tenofovir Alafenamide; PI: Protease Inhibitors; INSTI: Integrase Strand Transfer Inhibitors
