# Supplementary material for: Beyond HIV: investigating key drivers of metabolic syndrome in midlife women with HIV in the INSTI era
Source: AIDS Res Ther. 2026 Jul 1;23:92. doi: 10.1186/s12981-026-00913-1 (PMC13625421; doi:10.1186/s12981-026-00913-1)
Supplement: Supplementary file 3 [file 12981_2026_913_MOESM3_ESM.docx]

**Table S3.** Bivariate and Multivariate Cox Regression Analysis of factors associated with hyperglycemia.

| Covariates | Bivariate O.R. (95% C.I., p-value) | Multivariate O.R. (95% C.I., p-value) |
| --- | --- | --- |
| Age≥ 60years | 2.48 (1.02-6.10, p=0.04) | 1.42 (0.42-4.77, p=0.57) |
| Menopause* | 3.59 (1.14-15.98, p=0.05) | 1.64 (0.36-9.11, p=0.54) |
| Physical activity ** | 0.22 (0.05-0.69, p=0.02) | **0.19 (0.04-0.72, p=0.03)** |
| Arduous work*** | 2.34 (0.96-5.99, p=0.07) | - |
| Active/former smoker | 0.77 (0.31-1.83, p=0.55) | - |
| Alcohol abuse**** | 0.50 (0.03-2.98, p=0.52) | - |
| Dyslipidemia (family history) | 6.00 (0.94-47.60, p=0.06) | - |
| PWID | 4.01 (0.74-74.67, p=0.19) | - |
| ≥10 Years of HIV | 5.52 (1.05-101.93, p=0.10) | - |
| ≥10 Years of ART | 3.06 (1.18-8.99, p=0.03) | 2.69 (0.83-10.00, p=0.11) |
| CD4 nadir <200 cells/mm3 | 1.94 (0.80-4.71, p=0.14) | - |
| ≥5 Years of TAF use | 3.38 (1.34-8.57, p=0.009) | 1.47 (0.43-4.79, p=0.53) |
| ≥10 Years of PI use | 5.52 (1.77-17.71, p=0.003) | **5.73 (1.33-27.62, p=0.02)** |
| ≥10 Years of INSTI use | 2.48 (1.02-6.10, p=0.04) | 1.42 (0.42-4.77, p=0.57) |

** Defined as the absence of menstruation for more than 12 months, as reported by patients.*

*** Engagement in any leisure-time physical exercise or activity performed at least once per week for a minimum duration of 60 consecutive minutes as reported by patients.*

****More than 1 standard drink per day (i.e., >10–12 grams of pure ethanol per day) as reported by patients*

****** *Farm laborer, domestic worker, factory laborer, healthcare assistant, nurse, factory worker, hotel/hospital cleaner.*

O.R.: Odds Ratio; C.I.: Confidence Interval; HIV: Human Immunodeficiency Virus; PWID: People Who Inject Drugs; AIDS: Acquired Immunodeficiency Syndrome; HBsAg: Hepatitis B Surface Antigen; HCVAb: Hepatitis C Virus Antibodies; ART: Antiretroviral Therapy; TAF: Tenofovir Alafenamide; PI: Protease Inhibitors; INSTI: Integrase Strand Transfer Inhibitors
