# Supplementary material for: Beyond HIV: investigating key drivers of metabolic syndrome in midlife women with HIV in the INSTI era
Source: AIDS Res Ther. 2026 Jul 1;23:92. doi: 10.1186/s12981-026-00913-1 (PMC13625421; doi:10.1186/s12981-026-00913-1)
Supplement: Supplementary file 4 [file 12981_2026_913_MOESM4_ESM.docx]

**Table S4.** Bivariate and Multivariate Cox Regression Analysis of factors associated with an abdominal circumference >88 cm.

| Covariates | Bivariate O.R. (95% C.I., p-value) | Multivariate O.R. (95% C.I., p-value) |
| --- | --- | --- |
| Age≥ 60years | 2.19 (0.99-5.00, p=0.06) | - |
| Menopause* | 1.35 (0.56-3.32, p=0.50) | - |
| Physical activity ** | 0.38 (0.16-0.86, p=0.02) | **0.30 (0.11-0.77, p=0.01)** |
| Arduous work*** | 3.27 (1.05-12.39, p=0.05) | - |
| Active/former smoker | 0.93 (0.44-1.98, p=0.85) | - |
| Alcohol abuse**** | 2.45 (0.50-17.67, p=0.30) | - |
| Dyslipidemia (family history) | 1.01 (0.47-2.15, p=0.99) | - |
| PWID | 0.93 (0.11-7.95, p=0.94) | - |
| ≥10 Years of HIV | 1.59 (0.47-5.69, p=0.46) | - |
| ≥10 Years of ART | 1.67 (0.59-4.96, p=0.34) | - |
| CD4 nadir <200 cells/mm3 | 0.83 (0.38-1.80, p=0.64) | - |
| ≥5 Years of TAF use | 1.05 (0.48-2.33, p=0.90) | - |
| ≥10 Years of PI use | 1.77 (0.75-4.32, p=0.20) | - |
| ≥10 Years of INSTI use | 3.27 (1.05-12.39, p=0.05) | - |

** Defined as the absence of menstruation for more than 12 months, as reported by patients.*

*** Engagement in any leisure-time physical exercise or activity performed at least once per week for a minimum duration of 60 consecutive minutes as reported by patients.*

****More than 1 standard drink per day (i.e., >10–12 grams of pure ethanol per day) as reported by patients*

****** *Farm laborer, domestic worker, factory laborer, healthcare assistant, nurse, factory worker, hotel/hospital cleaner.*

O.R.: Odds Ratio; C.I.: Confidence Interval; HIV: Human Immunodeficiency Virus; PWID: People Who Inject Drugs; AIDS: Acquired Immunodeficiency Syndrome; HBsAg: Hepatitis B Surface Antigen; HCVAb: Hepatitis C Virus Antibodies; ART: Antiretroviral Therapy; TAF: Tenofovir Alafenamide; PI: Protease Inhibitors; INSTI: Integrase Strand Transfer Inhibitors
