# Supplementary material for: Beyond HIV: investigating key drivers of metabolic syndrome in midlife women with HIV in the INSTI era
Source: AIDS Res Ther. 2026 Jul 1;23:92. doi: 10.1186/s12981-026-00913-1 (PMC13625421; doi:10.1186/s12981-026-00913-1)
Supplement: Supplementary file 5 [file 12981_2026_913_MOESM5_ESM.docx]

**Table S5.** Bivariate and Multivariate Cox Regression Analysis of factors associated with HDL-cholesterol levels <50 mg/dL.

| Covariates | Bivariate O.R. (95% C.I., p-value) | Multivariate O.R. (95% C.I., p-value) |
| --- | --- | --- |
| Age≥ 60years | 1.40 (0.62-3.11, p=0.41) | **-** |
| Menopause* | 1.15 (0.49-2.82, p=0.75) | **-** |
| Physical activity ** | 1.12 (0.49-2.51, p=0.78) | **-** |
| Arduous work*** | 2.47 (1.13-5.54, p=0.02) | **4.78 (1.83-13.73, p=0.002)** |
| Active/former smoker | 0.79 (0.36-1.70, p=0.55) | **-** |
| Alcohol abuse**** | 1.27 (0.25-5.45, p=0.75) | **-** |
| Dyslipidemia (family history) | 2.33 (1.07-5.18, p=0.03) | **4.06 (1.56-11.61, p=0.006)** |
| PWID | 3.29 (0.52-25.81, p=0.20) | **-** |
| ≥10 Years of HIV | 0.43 (0.14-1.36, p=0.14) | **-** |
| ≥10 Years of ART | 0.72 (0.26-2.11, p=0.53) | **-** |
| CD4 nadir <200 cells/mm3 | 1.28 (0.59-2.83, p=0.53) | **-** |
| ≥5 Years of TAF use | 0.90 (0.40-1.98, p=0.80) | **-** |
| ≥10 Years of PIs use | 1.20 (0.50-2.81, p=0.68) | **-** |
| ≥10 Years of INSTIs use | 0.28 (0.04-1.10, p=0.11) | **-** |

** Defined as the absence of menstruation for more than 12 months, as reported by patients.*

*** Engagement in any leisure-time physical exercise or activity performed at least once per week for a minimum duration of 60 consecutive minutes as reported by patients.*

****More than 1 standard drink per day (i.e., >10–12 grams of pure ethanol per day) as reported by patients*

****** *Farm laborer, domestic worker, factory laborer, healthcare assistant, nurse, factory worker, hotel/hospital cleaner.*

O.R.: Odds Ratio; C.I.: Confidence Interval; HIV: Human Immunodeficiency Virus; PWID: People Who Inject Drugs; AIDS: Acquired Immunodeficiency Syndrome; HBsAg: Hepatitis B Surface Antigen; HCVAb: Hepatitis C Virus Antibodies; ART: Antiretroviral Treatment; TAF: Tenofovir Alafenamide; PI: Protease Inhibitors; INSTI: Integrase Strand Transfer Inhibitors
